# Pathological and biochemical evaluation of radish microgreen on diabetes and aflatoxicosis in rats

**DOI:** 10.1007/s11356-023-29334-7

**Published:** 2023-08-22

**Authors:** Sara M. Mohamed, Tahany A. A. Aly, Marwa S. Khattab, Emam A Abdel-Rahim, Ammar AL-Farga

**Affiliations:** 1 Regional Center for Food and Feed, Agriculture Research Center, Ministry of Agriculture, Giza, Egypt; 2 Pathology Department, Faculty of Veterinary Medicine, Cairo University, Giza Square, Giza, 12211 Egypt; 3 Biochemistry Department, Faculty of Agriculture, Cairo University, Giza, Egypt; 4 Department of Biochemistry, College of Sciences, University of Jeddah, Jeddah, Saudi Arabia

**Keywords:** Radish microgreen, Diabetes, Aflatoxin, Oxidative stress, Pancreas

## Abstract

Diabetes mellitus type 2 remains one of the common diseases nowadays. Several risk factors can be implicated like increased environmental pollution. This study is aimed at evaluating the toxic effect of aflatoxin on diabetes mellitus and possible protection using natural food like radish microgreen (RM). Forty-eight male rats were randomly assigned to 8 groups: G1 control group, G2 RM group, G3 aflatoxin group, G4 aflatoxin-RM group, G5 diabetic group, G6 diabetic RM group, G7 diabetic–aflatoxin group, G8 diabetic, aflatoxin, RM group. Phytane and citronellyl tiglate were the main phytochemicals present in RM. The glucose and insulin levels were the worst in G5 and G7 groups. RM feeding restored glucose level to normal but did not alter insulin level. Insulin resistance was decreased, and insulin sensitivity was increased in groups fed RM. Liver and kidney function parameters and LDH activity were improved in groups fed RM. Histopathology of the pancreas and immunohistochemistry of insulin in pancreatic islets was improved in groups fed RM. In RM fed groups, the MDA content was decreased, whereas GSH content and antioxidant enzymes activity were increased. In conclusion, feeding RM in diabetic and/or aflatoxicated groups improved all evaluated parameters which could be due to its antioxidant potential.

## Introduction

Many chronic diseases like diabetes mellitus type 2 became prevalent in recent years. A lot of risk factors are being investigated for their potential role in the development of such diseases (Fletcher et al. 2003). Diabetes mellitus has a deleterious effect on many organs including retinopathy, nephropathy, and/or neuropathy (Emerging Risk Factors Collaboration et al. 2010). The mechanism of damage in DM was mainly due to the overproduction of free radicals which results in oxidative stress and cell injury (Giacco and Brownlee 2010). Aflatoxin is a toxic carcinogen, and mutagen is a common food contaminant due to inefficient food preservation (Bennett and Klich 2003). Aflatoxin B1 (AFB1) mediates its toxicity mainly by oxidative stress and AFB1-Exo 8,9-epoxide metabolite produced during its metabolism (Benkerroum 2020). The association of aflatoxicosis and diabetes mellitus was referred to in previous studies which indicated the aggravation of DM adjunct with a low dose of aflatoxin (Mohamed et al. 2022).

Diet modification with natural food rich in antioxidants would help significantly in lowering the injurious effect of DM and aflatoxicosis. Red radish “*Raphanus sativus*” family “*Brassicaceae*” is a radical crop that is eaten raw, pickled, or cooked (Otsuki et al. 2004; Tamura et al. 2010). Red radish is rich in important vitamins and minerals (folic acid), anthocyanins, potassium (K), calcium (Ca), copper (Cu), iron (Fe), phosphorus (P), zinc (Zn), anthocyanins, fiber, and antioxidants making it a potential candidate in avoiding cardiovascular diseases, dyslipidemic conditions, and hypoglycemic influences (Kim et al. 2014; Anna et al. 2016; Khedr and El Sheikh 2016). Vivarelli et al. (2016) suggested that radish juice could be a good nutraceutical product with efficient antioxidant, hypolipidemic, and anti-obesity effects.

Therefore, this study was done to evaluate the effect of radish microgreen on insulinemia, biochemical alteration, oxidative stress, and histopathological lesions induced by DM and/or aflatoxicosis.

## Materials and methods

### Microgreens of radish

Its microgreens (RM) grown in an open field and harvested at the fully expended green cotyledons stage which was 14 days from seed soaking, washing, and hulling (Abdallah 2008). Harvested RM was air-dried for 3 days according to previous study (Dzowela et al. 1995) and ground into powder. The phytochemical compounds present in RM powder were determined according to a previous method using gas chromatography-mass spectrometry GC/MS technique (Santana et al. 2013). The analysis was conducted using a GC (Agilent Technology 7890A) coupled with a mass selective detector (MSD, Agilent 7000 Triple Quad) equipped with Agilent HP-5 ms capillary column. The identification of components was based on a comparison of their mass spectra with the authentic compounds and by computer matching with the NIST library as well as by comparison of the fragmentation pattern of the mass spectral data with those registered in the literature. All local, national, or international guidelines and legislation were adhered to use of plants in this study.

### Aflatoxin preparation

*Aspergillus flavus* strain (NRRL 3357) (laboratory of mycotoxin, National Research Center, Dokki, Cairo) was grown on slants of prepared Czapek’s agar media (Davis et al. 1966) and incubated at 25–29 °C for 9 days. Then, the 9-day-old *Aspergillus flavus* spores were incubated in a cooled sterilized flask containing prepared liquid yeast medium for 9 days at 25–29 °C. The filtrate was stored in tightly wrapped bottles in aluminum foil at 4 °C. Based on the Association of Official Analytic Chemists (AOAC) method, total aflatoxin concentration was measured using a slightly modified immunoaffinity method (Trucksess et al. 1991) in a pre-calibrated VICAM Series-4 fluorometer set at 360 nm excitation and 450 nm emission. Aflatoxin was recognized by a modification of the HPLC – AFLATEST procedure Agilent 1200 series USA (HPLC equipment with two pumps, column C18, Lichrospher 100 RP-18).

### Animals

Male rats were purchased from the National Research Center (El Dokki, El Giza, Egypt), housed in plastic cages (3 rats per cage), and acclimated for two weeks. Animals were had free access to water and fed a pelleted diet. The temperature and relative humidity were adjusted at 25 ± 2 °C, 50–60%, respectively. This study was granted ethical approval by the Institutional Animal Care and Use Committee, Faculty of Veterinary Medicine, Cairo University (Vet CU01102020224). The study is reported in accordance with ARRIVE guidelines.

### Induction of type 2 diabetes mellitus

To induce diabetes, rats were fed with a high-fat diet ad libitum for 2 weeks and then injected with a low dose of streptozotocin (STZ) (single dose of 30 mg kg^−1^) (Zhang et al. 2008). The fasting blood glucose levels of all the rats are measured after seven days. Diabetic rats with blood glucose levels ≥ 200 mg dL^−1^ were chosen for further experimentation.

### Diets and their preparation

Four different diets were formulated: a control diet according to the AIN-76, a radish microgreen (RM) diet with 10% RM powder replacing corn starch, an HFD with 20% palm oil, and an HF and RM diet with 20% palm oil and 10% RM (Table 1). Diets were manufactured into pellets.

**Table 1:** Composition of diets

Ingredients	Control diet	RM diet	High-fat diet	High-fat and RM diet
Casein	20.0	20.0	20.0	20.0
Corn starch	65.0	55.0	50.0	40.0
Mineral mix^2^	3.5	3.5	3.5	3.5
Vitamin mix^2^	1.0	1.0	1.0	1.0
DL-methionine	0.3	0.3	0.3	0.3
Choline bitartrate	0.2	0.2	0.2	0.2
Cellulose powder	5.0	5.0	5.0	5.0
Palm oil	5.0	5.0	20.0	20.0
Radish microgreen	-	10.0	-	10.0

^1^Weight percentage; ^2^based on AIN-76

### Experimental design

Forty-eight male albino rats were randomly assigned to 8 groups (6 rats each). G1 is a normal control. G2 rats were fed an RM diet. G3 rats were given aflatoxin (30 μg/kg) 3 days/week orally. G4 rats were given aflatoxin and fed RM. G5 is diabetic rats fed a high-fat diet (HFD). G6 is diabetic rats fed HFD with RM. G7 is diabetic rats fed HFD and given aflatoxin. G8 is diabetic rats fed HFD with RM and administered aflatoxin orally. All rats were weighed at the beginning and end of the experiment to record the initial weight and final weight, respectively. Bodyweight (BW) = final weight-initial weight. Bodyweight gain was calculated by the following equation: BWG/100 g = BW/initial weight * 100. The animals were euthanized after 6 weeks.

### Determination of glucose level in plasma

Glucose level measured in plasma by enzymatic and calorimetric method (Trinder 1969) in which glucose oxidase (GOD) catalyzed the oxidation of glucose to gluconic acid.

### Determination of serum insulin hormone

A rat insulin ELISA (Thermo Fisher Scientific, Waltham, MA) was used to measure serum insulin according to manufacturer protocol (Temple et al. 1992). An ELISA reader was used to read the plate at 450 nm. A standard curve was constructed using the absorbance values obtained for the standards against the insulin concentration on a log-log paper. Using the standard curve, the insulin concentration of the samples was calculated.

$$\mathrm{HOMA}-\mathrm{IR}=\;\left[\mathrm{fasting}\;\mathrm{insulin}\;\left(\mathrm{mU}/\mathrm L\right)\;\times\;\mathrm{fasting}\;\mathrm{glucose}\;\left(\mathrm{mg}/\mathrm{dL}\right)\;\times\;0.0555\right]/\;22.5$$ 

$$\mathrm{HOMA}-\%\mathrm B\;=\;\left[20\;\times\;\mathrm{fasting}\;\mathrm{insulin}\;\left(\mathrm{mU}/\mathrm L\right)\right]\;/\;\left[\mathrm{fasting}\;\mathrm{glucose}\;\left(\mathrm{mg}/\mathrm{dL}\right)\;\times\;0.055)-3.5\right]$$ 

$$\mathrm{HOMA}-\%\mathrm S\left[1/\mathrm{HOMA}-\mathrm{IR}\right]\times100$$ 

$$\mathrm{Disposition}\;\mathrm{index}\;1=\;\left(\mathrm{HOMA}-\%\mathrm S/100\right)\times\left(\mathrm{HOMA}-\%\mathrm B/100\right)$$ 

### Determination of liver function

Serum AST, ALT, and ALP activity in addition to total bilirubin and total protein concentration were determined based on previous methods (Reitman and Frankel 1957; Belfield and Goldberg 1971; Walter and Gerarde 1970; Gornnall et al. 1949).

### Determination of kidney function parameters and LDH

Serum lactate dehydrogenase activity (LDH) and serum urea and creatinine were estimated following previous methods (Naithani and Singh 2006; Fawcett and Scott 1960; Moore and Sharer 2017).

### Histopathology

Specimen of pancreas, liver, and kidneys of rats was fixed in 10% neutral buffered formalin and processed by paraffin embedding technique. Tissue sections 4-μm-thick were stained by hematoxylin and eosin stain, examined by light microscopy, and photographed using a digital camera (Olympus XC30, Tokyo, Japan).

### Immunohistochemistry

Anti-insulin antibodies (Invitrogen, Thermo-Fisher Scientific, USA) and the avidin-biotin-peroxidase complex according to kit manufacturer protocol (Dako, North America, Inc., MI, USA) were used to detect insulin in paraffin-embedded tissue sections of the pancreas. 3,3′-diaminobenzidine was used as a substrate. Image J software was used to measure the area % of positive insulin in beta cells of the pancreatic islets in three photos/rats in each group at a 400× magnification power.

### Determination of oxidative stress in liver

Reduced glutathione (GSH), malondialdehyde (MDA), superoxide dismutase (SOD) activity, and catalase (CAT) activity were assessed in liver homogenate following previous methods (Beutler et al. 1963; Nishikimi et al. 1972; Ohkawa et al. 1979; Aebi 1984).

### Statistical analysis

A statistical package for the social science program version was used to detect standard deviation and standard error (Kinnear and Gray 1999). A significant difference between means of treatment was detected by LSD (least significant difference) (Waller and Duncan 1969). The area % of insulin-positive cells was evaluated for homogeneity and analyzed by the ANOVA test followed by Tamahne’s test and Duncan’s test to detect significance between groups.

## Results and discussion

RM was found to contain phytane as the major natural phytochemical (38.27%) which is one of the essential oils possessing an antioxidant property (Table 2) (Kamali et al. 2015). Citronellyl tiglate was also reported to have antibacterial, antifungal, antioxidant, and anti-inflammatory properties (Cavar and Maksimovic 2012). Moreover, squalene (SQ), a natural compound, is a precursor of various hormones in animals and sterols in plants which reduces skin damage by UV radiation, LDL levels, and cholesterol in the blood, in addition to preventing cardiovascular diseases (Lozano-grande et al. 2018).

**Table 2:** Phytochemical compounds identified in the ethanolic extract fractionation of Egyptian radish microgreen by GC-MS

No	R.T	Compound name	Area sum %
1	9.23	2-Hexadecanol	0.42
2	12.29	Hexa-hydro-farnesol	0.7
3	12.40	Phytanic acid	3.68
4	12.61	Phytol	0.64
5	12.77	Phytol, acetate	1.83
6	13.37	Stigmasterol acetate	0.61
7	13.43	6,4′-Dimethoxy-7-hydroxyisoflavone	4.32
8	13.56	4′,6-Dimethoxyisoflavone-7-O-β-D-glucopyranoside	0.75
9	13.76	7,3′-Dimethoxy-3-hydroxyflavone	5.49
10	13.86	4′-Acetoxy-7-hydroxy-6-methoxyisoflavone	0.5
11	14.35	9-cis-Retinoic acid	2.46
12	14.62	Isolongifolol	0.46
13	14.74	Citronellyl tiglate	9.89
14	15.57	Geranyl isovalerate	0.47
15	16.24	1-Dodecanol, 3,7,11-trimethyl	0.99
16	17.50	Tetradecanethiol	0.68
17	17.68	Squalane	3.39
18	18.12	β-Citronellol	0.45
19	18.96	Crocetane	1.28
20	20.04	Farnesan	1.53
21	20.49	Norphytan	14.97
22	20.85	3-(3,4-Dimethoxyphenyl)-4-methylcoumarin	0.46
23	21.56	Ascorbic acid	5.76
24	22.85	Phytane	38.27

### Bodyweight gain

The body weight gain (BWG) of rats was the least in the STZ-aflatoxin group followed by the STZ-aflatoxin-RM group, STZ group, and STZ-RM group. The injection with STZ in rats as recorded previously results in a severe decrease in body weight (Zafar and Naqvi 2010). The BWG in the AF group was also reduced compared to the control; however, it was partially restored in the aflatoxin-RM group. The highest BWG was recorded in the RM group which might be due to the beneficial effect of radish microgreen on body metabolism (Table 3).

**Table 3:** Body weight gain of rats at the end of 6-week experimental period

Treatment	Initial weight (G)	Final weight (g)	BWG/L % of control (G1)
G1 control	118.8 ± 8.91	200.57 ± 10.94	100
G2 Radish	111.42 ± 8.71	199.70 ± 16.14	115
G3 aflatoxin	124.40 ± 14.09	168.78 ± 6.35	52
G4 aflatoxin RM	124.83 ± 8.07	197.05 ± 13.50	84
G5 STZ	124.80 ± 14.39	132.27 ± 2.77	9
G6 STZ-RM	119.37 ± 14.05	128.49 ± 13.71	11
G7 STZ-aflatoxin	135.73 ± 18.69	93.33 ± 17.68	−45
G8 STZ-aflatoxin-RM	129.21 ± 11.01	101.60 ± 0.48	−31

*RM* radish microgreen, *BW* body weight, *BWG* body weight gain. All values are represented as mean ± S.D. Means with different letters superscripts are significantly different (*p* < 0.05)

### Blood glucose and insulin level

The glucose level was increased significantly in rats of all groups receiving aflatoxin and/or injected with STZ compared to control and RM groups. The highest significant increase in glucose level was in diabetic rats fed aflatoxin. However, the glucose level was decreased significantly in the groups fed RM compared to their counterparts not fed RM. The hypoglycemic effect of radish was attributed to the insulin-like components (e.g., polyphenolic substances or glucosidase-inhibiting components present in its water-soluble extract) (Taniguchi et al. 2007).

The insulin level was significantly reduced in the groups injected with STZ, especially in the aflatoxin-STZ group compared to the control and RM group, and slightly decreased in groups fed aflatoxin (G3 and G4). Feeding RM did not improve the insulin level significantly in treated groups (G4, G6, and G8) (Table 4). This indicates that radish microgreen reversed the hyperglycemia without increasing the insulin secretion. The radish was believed to have an anti-diabetic property as it endorses glucose uptake and energy metabolism, reduces glucose absorption in the intestine, and improves the hormonal-induced glucose hemostasis (Banihani 2017).

**Table 4:** Blood glucose and insulin levels in different experimental animal groups at the end of the experimental period

Groups	Treatment	Blood glucose		Blood insulin	
		mg/dL	m mol/dL	mu/L	ng/L
G1	Control	100 ± 8.1^e^	5.56 ± 0.45^e^	7.37 ± 0.44^a^	1.84 ± 0.08^a^
G2	RM	99 ± 9.0^e^	5.50 ± 0.50^e^	7.53 ± 0.51^a^	1.88 ± 0.09^a^
G3	Aflatoxin	131 ± 11.1^c^	7.28 ± 0.62^c^	6.67 ± 0.42^ab^	1.67 ± 0.08^ab^
G4	Aflatoxin RM	114 ± 10.2^d^	6.33 ± 0.57^d^	6.89 ± 0.33^ab^	1.72 ± 0.09^ab^
G5	STZ	150 ± 8.2^b^	8.33 ± 0.46^b^	6.20 ± 0.41^b^	1.55 ± 0.08^b^
G6	STZ RM	126 ± 7.7^cd^	7.00 ± 0.43^cd^	6.33 ± 0.32^b^	1.58 ± 0.09^b^
G7	STZ aflatoxin	176 ± 8.3^a^	9.78 ± 0.46^a^	5.70 ± 0.39^b^	1.43 ± 0.09^b^
G8	STZ, aflatoxin, RM	148 ± 9.2^b^	8.22 ± 0.51^b^	5.80 ± 0.30^b^	1.45 ± 0.10^b^

*RM* radish microgreen. All values are represented as mean ± S.D. Means with different letters are significantly different (*p* < 0.05)

### Homeostatic model assessment

The validation of HOMA-IR to determine insulin-resistance in Wistar rats was assured previously (Antunes et al. 2016). In the current study, β-cell function was significantly decreased in groups injected with STZ and/or fed aflatoxin, especially in the aflatoxin-STZ group. On the other hand, the β-cell function was significantly restored partly in the groups fed RM. The insulin resistance was significantly increased in the groups injected with STZ and/or fed aflatoxin, whereas it was comparable to control in the groups fed RM (G4 and G6). Insulin sensitivity was significantly decreased in the STZ group especially in the STZ-aflatoxin group, whereas it was improved in the groups fed RM with aflatoxin and/or injected with STZ and became comparable to control. The deposition index in groups injected with STZ and/or fed aflatoxin was significantly decreased particularly in the STZ-aflatoxin group. In contrast, RM feed significantly increased the deposition index in groups fed aflatoxin with or without STZ injection (Table 5). These results suggests that radish microgreen has an antidiabetic property which is mainly due the amelioration of insulin sensitivity and not by increasing insulin secretion as indicated in previous research (Taniguchi et al. 2006).

**Table 5:** Homeostatic model assessment of β-cell function (HOMA-B), homeostatic model assessment of insulin resistance (HOMA-IR), homeostatic model assessment of insulin sensitivity (HOMA-% S), and disposition index (DI) of the experimental rats

Groups	Treatment	HOMA-B	HOMA-IR	HOMA-%S	Disposition index
		Value	Value	Value	value
G1	Control	71.55 ± 6.61^a^	1.82 ± 0.12^c^	54.95 ± 4.41^a^	0.393 ± 0.031^a^
G2	RM	75.30 ± 7.02^a^	1.84 ± 0.18^c^	54.35 ± 4.52^a^	0.409 ± 0.032^a^
G3	Aflatoxin	35.29 ± 3.12^c^	2.16 ± 0.20^b^	47.30 ± 4.18^ab^	0.167 ± 0.011^c^
G4	Aflatoxin RM	48.62 ± 3.31^b^	1.94 ± 0.16^c^	51.55 ± 4.82^a^	0.251 ± 0.018^b^
G5	STZ	25.67 ± 2.41^d^	2.30 ± 0.19^b^	43.48 ± 4.34^b^	0.112 ± 0.010^d^
G6	STZ-RM	36.17 ± 2.98^c^	1.97 ± 0.18^bc^	50.76 ± 4.67^a^	0.184 ± 0.014^c^
G7	STZ-aflatoxin	18.15 ± 1.12^e^	2.48 ± 0.21^a^	40.32 ± 3.98^b^	0.073 ± 0.006^e^
G8	STZ-aflatoxin-RM	24.58 ± 2.13^d^	2.12 ± 0.20^b^	47.17 ± 4.13^ab^	0.116 ± 0.012^d^

*RM* radish microgreen. All values are represented as mean ± S.D. Means with different letters are significantly different (*p* < 0.05)

### Liver function parameters

The liver function enzymes and total bilirubin were significantly increased, whereas total protein was significantly decreased in groups receiving aflatoxin and/or STZ with the highest increase in G7 (aflatoxin and STZ). On the other side, diabetic and/or aflatoxicosis rats fed RM (G4, G6, and G8) showed a significant decrease in liver function enzymes compared to their counterparts. Total bilirubin was also decreased significantly, and total protein was significantly increased in treated groups except for G4 (aflatoxin-RM) (Table 6). A previous study also showed that radish improved the liver function parameters in rats (Lee et al. 2012).

**Table 6:** Liver function enzymes, total bilirubin, and total protein in different groups

Treatment	AST(U/L)	ALT (U/L)	ALP (U/L)	Bilirubin total (mg/dL)	T.protein (g/dL)
G1 control	124.83 ± 10.12^e^	64.83 ± 5.77^c^	722.50 ± 20.26^d^	0.51 ± 0.07^d^	6.86 ± 0.09^a^
G2 RM	121.11 ± 9.23^e^	53.17 ± 4.75^c^	782.50 ± 80.24^d^	0.53 ± 0.07^cd^	7.35 ± 0.18^a^
G3 aflatoxin	247.67 ± 9.29^ab^	93.17 ± 5.84^b^	1268.67 ± 101.54^a^	0.71 ± 0.09^b^	6.43 ± 0.13^b^
G4 aflatoxin RM	143.33 ± 1.61^d^	78.00 ± 2.65^b^	884.00 ± 73.74^c^	0.80 ± 0.22^ab^	6.13 ± 0.74^b^
G5 STZ	189.50 ± 6.06^b^	71.83 ± 2.84^bc^	980.50 ± 17.50^b^	0.77 ± 0.16^ab^	6.13 ± 0.28^b^
G6 STZ- radish	152.17 ± 4.86^cd^	52.17 ± 3.25^c^	805.33 ± 53.46^c^	0.62 ± 0.15^c^	6.63 ± 0.50^ab^
G7 STZ-aflatoxin	322.00 ± 12.12^a^	125.00 ± 6.06^a^	1303.67 ± 117.27^a^	0.89 ± 0.02^a^	5.86 ± 0.95^b^
G8 STZ- aflatoxin RM	174.33 ± 4.62^c^	79.33 ± 2.08^b^	882.33 ± 75.08^c^	0.57 ± 0.10^c^	5.98 ± 0.31^b^

All values are represented as mean ± S.D. Means with different letters are significantly different (*p* < 0.05)

### Kidney function parameters and LDH activity

Rats receiving aflatoxin and/or STZ showed increased LDH activity with the highest increase in G7. Urea and creatinine concentrations were significantly elevated in aflatoxicated groups (G3 and G7). Feeding RM to diabetic and/or aflatoxicated rats decreased significantly creatinine and urea concentration in G4 and G8 and LDH activity in G4, G6, and G8 (Table 7). It was also reported before that radish extract improved the kidney function parameters due to toxicity (Salah-Abbès et al. 2008).

**Table 7:** Kidneys function enzymes and lactate dehydrogenase (LDH) in different groups

Treatment	LDH activity (U/L)	Urea (mg/dL)	Creatinine (mg/dL)
G1 control	1388.50 ± 29.50^e^	39.67 ± 1.44^b^	0.61 ± 0.11^c^
G2 RM	1292.83 ± 55.65^e^	41.17 ± 4.04^b^	0.74 ± 0.01^b^
G3 aflatoxin	2136.67 ± 91.12^b^	52.17 ± 9.17^a^	0.81 ± 0.19^a^
G4 aflatoxin-RM	1754.00 ± 5.77^c^	52.33 ± 4.50^a^	0.74 ± 0.14^b^
G5 STZ	1743.17 ± 58.31^c^	40.67 ± 2.13^b^	0.71 ± 0.08^b^
G6 STZ- RM	1429.50 ± 38.15^d^	37.00 ± 3.50^b^	0.71 ± 0.12^b^
G7 STZ-aflatoxin	2426.00 ± 77.74^a^	41.00 ± 2.00^b^	0.80 ± 0.06^a^
G8 STZ-aflatoxin-RM	1433.00 ± 36.68^d^	39.67 ± 0.58^b^	0.68 ± 0.25^c^

All values are represented as mean ± S.D. Means with different letters are significantly different (*p* < 0.05)

### Histopathological and immunohistochemical findings in pancreas

**Fig. 1:**
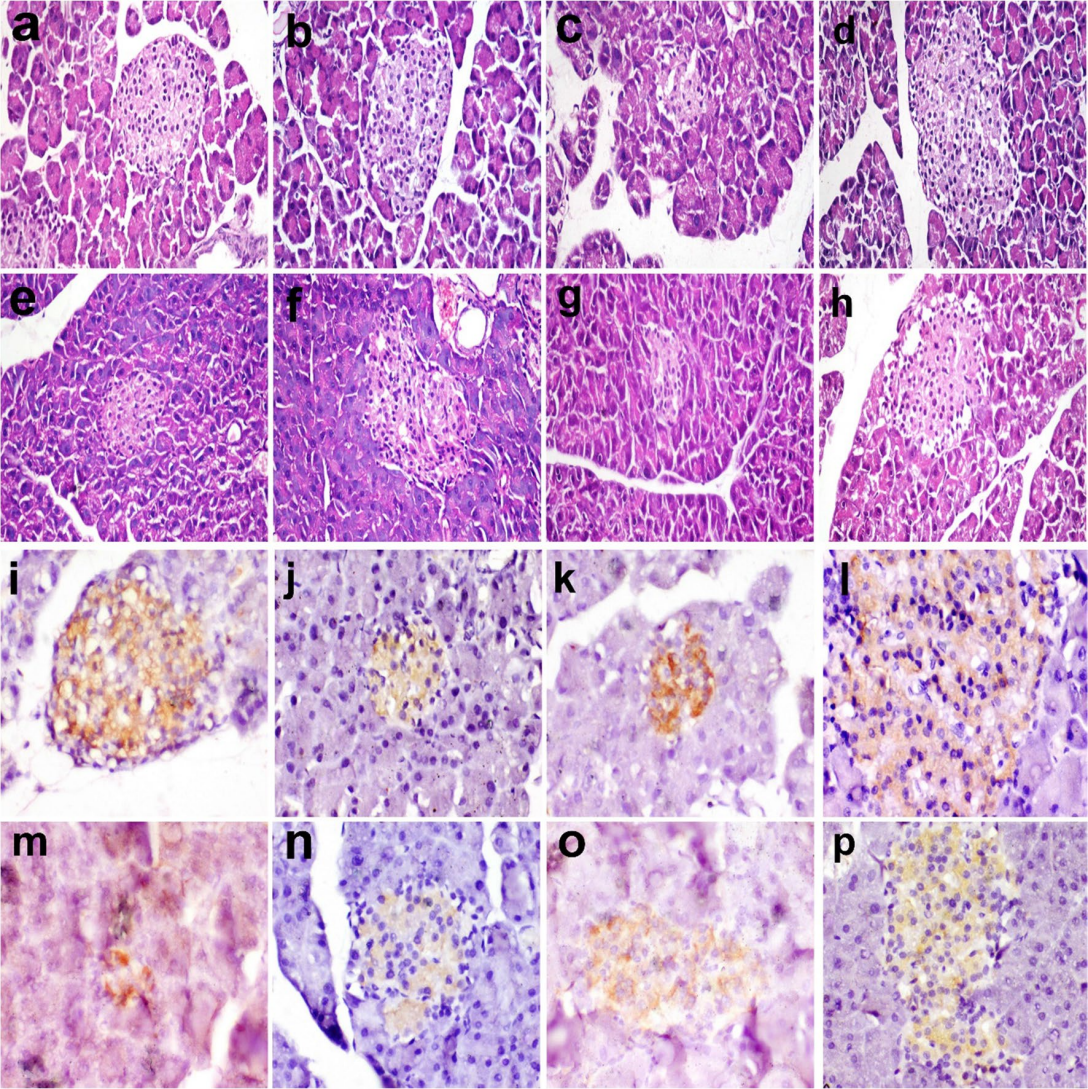
Pancreas of rats showing islets of Langerhans in **(a)** control group, **b** RM group, **c** aflatoxin group, **d** aflatoxin and RM group, **e** STZ group, **f** STZ and RM group, **g** STZ and aflatoxin group, and **h** STZ, aflatoxin, and BM. Hematoxylin and eosin stain (X200). **i–p** Pancreas of rats showing insulin-positive beta cells in islets of Langerhans in **(i)** control group, **j** RM group, **k** aflatoxin group, **l** aflatoxin and RM group, **m** STZ group, **n** STZ and RM group, **o** STZ and aflatoxin group, and **p** STZ, aflatoxin, and RM. Immunoperoxidase stain (×400)

In the control group (G1) and RM group (G2), pancreas microscopy of rats revealed normal islets of Langerhans and pancreatic acini (Fig. 1a and b). On the other hand, the islets of Langerhans were of moderate-size with irregular boundaries and show cellular degeneration (Fig. 1c). Aflatoxin causes oxidative stress which particularly adversely affects β cells as they have a low levels of antioxidant enzyme expression (Kaneto et al. 2005). Radish microgreen in G4 improved the histopathology of Langerhans’ islets compared to G3 (Fig. 1d). Previous studies showed that natural compounds can protect the pancreas against aflatoxin damage (Mohamed et al. 2022 ; Abdel-Latif et al. 2017). This could be due to the rich content of radish with antioxidant compounds which might have protected the β cells.

In G5 injected with STZ, the Langerhans islets were small, surrounded by fibrous tissue and showed cellular degeneration (Fig. 1e). Similar lesions were observed in pancreas after STZ injection. STZ induces pancreatic cytotoxicity damaging the beta cells responsible for insulin secretion and thus resulting in diabetes (Kulkarni et al. 2012).

These lesions were partially alleviated in G6 fed RM (Fig. 1f). In G7 rats (STZ-aflatoxin group), the islets of Langerhans showed similar lesions to those observed in G5 (Fig. 1g); however, in G8 fed RM as well the islets histopathology was improved (Fig. 1h). This could also be attributed to the high antioxidant compounds present in radish. Similar study also showed the protective effect radish microgreen on the pancreatic cytotoxicity induced by STZ (Aly et al. 2020).

**Fig. 2:**
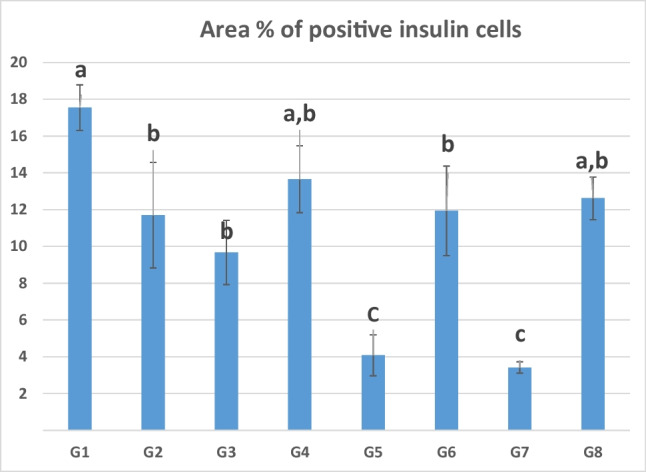
Area % of insulin-positive cells in the islets of Langerhans of the pancreas of different groups. G1: control group, G2: RM group, G3: aflatoxin group, G4: aflatoxin and RM group, G5: STZ group, G6: STZ and RM group, G7: STZ and aflatoxin group, and G8: STZ, aflatoxin and RM. Columns bearing different superscripts are significant at *p* < 0.05

In the control group (G1) and RM group (G2), the insulin-positive beta cells were organized in an ordered continuous cord in the islets of Langerhans, whereas the area % of beta cells was moderately reduced and the cells were arranged haphazardly in G3 (aflatoxin group) compared to the control group (Fig. 1i–p). In G4, the area % of beta cells was comparable to control. On the other hand, the area % of beta cells was significantly reduced in G5 (STZ) and G7 compared to control. In G6 and G8 fed RM, the area % of beta cells was comparable to control group (G1) (Fig. 2). Natural phytochemicals have an antioxidant property which prevents the damage of B cells (Mohamed et al. 2022; Abdel-Mobdy et al. 2021). Radish microgreen is one of the plants that is highly rich in these compounds as demonstrated in the present study.

**Fig. 3:**
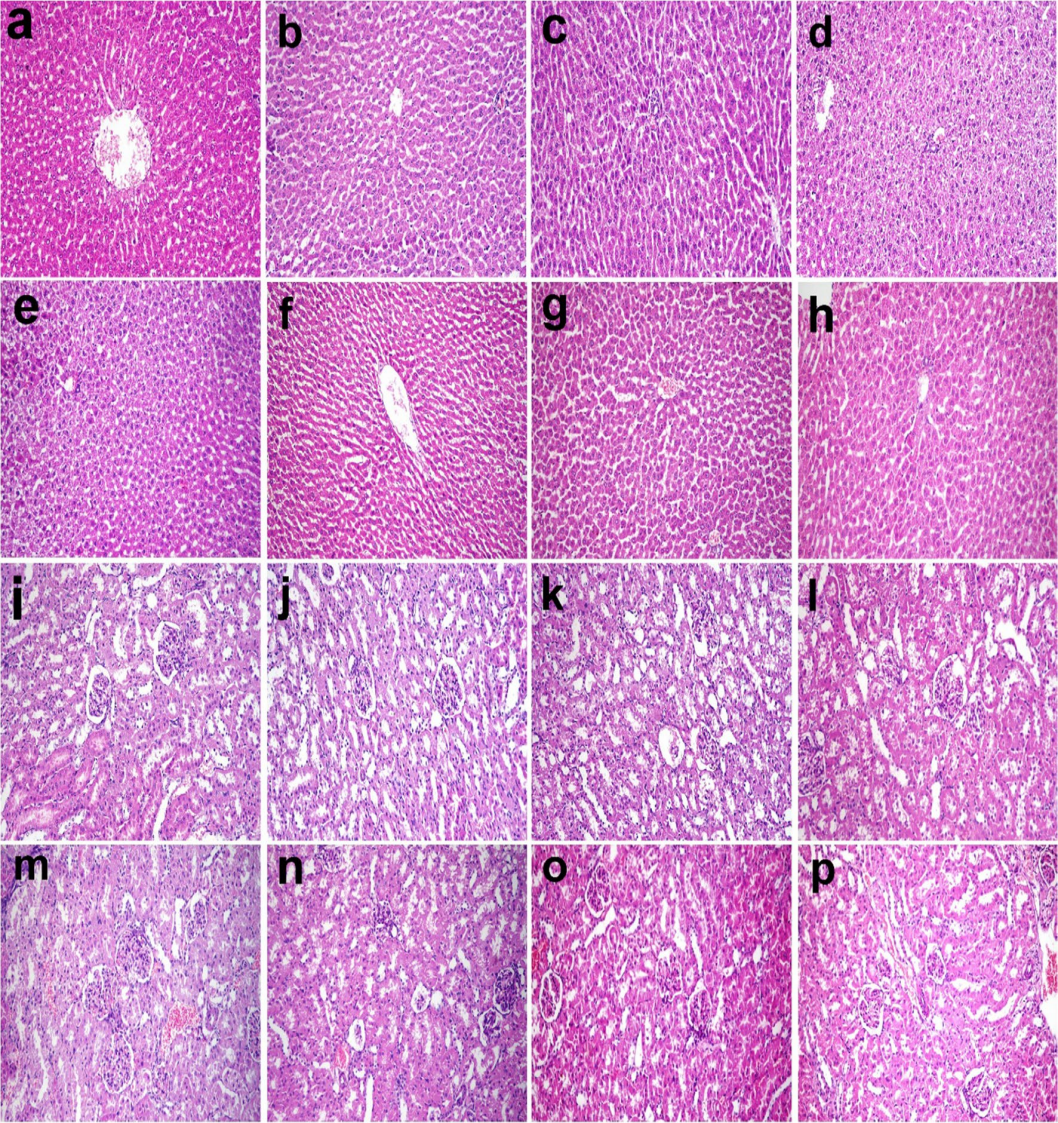
**a–h** histopathological structure of liver of rats in **(a)** control group, **b** RM group, **c** aflatoxin group, **d** aflatoxin and RM group, **e** STZ group, **f** STZ and RM group, **g** STZ and aflatoxin group, and **h** STZ, aflatoxin, and RM. **i–p** Kidney of rats in different groups **(i)** control group, **j** RM group, **k** aflatoxin group, **l** aflatoxin and RM group, **m** STZ group, **n** STZ and RM group, **o** STZ and aflatoxin group, and **p** STZ, aflatoxin, and RM. Hematoxylin and eosin stain (×200)

The liver was adversely affected with aflatoxin in which it had hypertrophied hepatocytes and mild solitary necrosis in addition to karyomegaly and binucleation. Feeding RM to rats in G4 decreased the severity of lesions compared to aflatoxicated groups. Mild periportal hepatocyte degeneration was observed in the liver of rats in G5 and G7 which was lessened in rats of G6 and G8 (Fig. 3a–h).

Microscopy of the kidneys revealed mild hypertrophy of tubular epithelium in G3 compared to control (G1) and RM (G2) which was relieved in the aflatoxin-BM group (G4). In STZ group (G5) and STZ-aflatoxin group (G7), the glomeruli showed increased glomerular matrix, mesangial cells hyperplasia, and mild thickening of glomerular basement membrane, whereas the tubular epithelium showed degenerative and necrotic changes with luminal casts. On the other hand, feeding RM to G6 and G8 rats improved the histopathological findings (Fig. 3i–p).

### Oxidative stress biomarkers in liver

The MDA content was increased, while GSH content was decreased significantly in the liver of rats in G7 (STZ-aflatoxin), G3 (aflatoxin), and G5 (STZ) consecutively. These groups also revealed a significantly decreased antioxidant enzymes activity compared to control. Contrariwise, feeding RM in G4, G6, and G8 significantly decreased the MDA content and increased significantly the GSH content and antioxidant enzymes activity (Table 8). Radish extract was documented to decrease the oxidative stress resulting from exposure to xenobiotics which is mainly due to its richness with antioxidant compounds (Salah-Abbès et al. 2008). The radish has a high content of isothiocyanate, kaempferol glycosides, and L-tryptophan compounds which protect the cells against lipid peroxidation and gets rid of free radicle intermediates of lipid peroxidation (Salah-Abbès et al. 2008; Sipos et al. 2002).

**Table 8:** Effect of radish microgreen on liver tissue peroxidation of rats

Treatment	MDA content (Nmol/g)	Liver oxidation system			
		GSH content (μmol/mL)	SOD activity (U/g)	CAT activity (U/g)	GST activity (μmol/gt)
G1 control	6.361 ± 0.421^de^	0.411 ± 0.032^a^	77.10 ± 8.88^a^	158.11 ± 7.81^a^	6.13 ± 0.43^a^
G2 radish	6.012 ± 0.366^e^	0.430 ± 0.029^a^	80.09 ± 6.67^a^	160.08 ± 10.00^a^	6.22 ± 0.48^a^
G3 aflatoxin	10.112 ± 0.712^b^	0.222 ± 0.013^d^	47.04 ± 3.71^d^	99.12 ± 6.16^cd^	4.11 ± 0.21^d^
G4 aflatoxin RM	8.001 ± 0.321^c^	0.300 ± 0.016^c^	52.03 ± 3.11^cd^	109.14 ± 6.34^c^	4.87 ± 0.40^b^
G5 STZ	8.221 ± 0.521^c^	0.301 ± 0.020^c^	60.09 ± 4.02^c^	120.12 ± 8.27^bc^	5.07 ± 0.32^b^
G6 STZ-RM	7.011 ± 0.411^d^	0.380 ± 0.019^b^	71.11 ± 5.01^b^	131.17 ± 7.86^b^	5.87 ± 0.31^a^
G7 STZ-aflatoxin	12.781 ± 0.941^a^	0.154 ± 0.010^e^	35.03 ± 2.12^e^	84.20 ± 5.14^e^	3.45 ± 0.21^e^
G8 STZ-aflatoxin RM	10.000 ± 0.836^b^	0.202 ± 0.013^d^	43.04 ± 3.21^**d**^	101.22 ± 7.50^cd^	4.01 ± 0.22^d^

All values are represented as mean ± S.D. Means with different letters are significantly different (*p* < 0.05)

In conclusion, feeding RM to diabetic and/or aflatoxicated rats decreased serum glucose level; ameliorated liver and kidney function parameters; improved histopathology of pancreas, liver, and kidneys; and improved liver oxidative stress parameters which could be due to its antioxidant potential.

## Data Availability

Samples of the compounds and data used during the current study are available from the corresponding author.
